# Revealing the Interactions Between Diabetes, Diabetes-Related Diseases, and Cancers Based on the Network Connectivity of Their Related Genes

**DOI:** 10.3389/fgene.2020.617136

**Published:** 2020-12-14

**Authors:** Lijuan Zhu, Ju Xiang, Qiuling Wang, Ailan Wang, Chao Li, Geng Tian, Huajun Zhang, Size Chen

**Affiliations:** ^1^College of Mathematics and Computer Science, Zhejiang Normal University, Jinhua, China; ^2^Neuroscience Research Center, Department of Basic Medical Sciences, Changsha Medical University, Changsha, China; ^3^School of Computer Science and Engineering, Central South University, Changsha, China; ^4^Department of Endocrinology, The Affiliated Yantai Yuhuangding Hospital of Qingdao University, Yantai, China; ^5^Geneis Beijing Co., Ltd., Beijing, China; ^6^Qingdao Geneis Institute of Big Data Mining and Precision Medicine, Qingdao, China; ^7^Department of Oncology, The First Affiliated Hospital of Guangdong Pharmaceutical University, Guangdong Provincial Engineering Research Center for Esophageal Cancer Precision Treatment, Guangzhou, China

**Keywords:** diabetes-related disease, PPI network, biological process, network connectivity, network modules

## Abstract

Diabetes-related diseases (DRDs), especially cancers pose a big threat to public health. Although people have explored pathological pathways of a few common DRDs, there is a lack of systematic studies on important biological processes (BPs) connecting diabetes and its related diseases/cancers. We have proposed and compared 10 protein–protein interaction (PPI)-based computational methods to study the connections between diabetes and 254 diseases, among which a method called DIconnectivity_eDMN performs the best in the sense that it infers a disease rank (according to its relation with diabetes) most consistent with that by literature mining. DIconnectivity_eDMN takes diabetes-related genes, other disease-related genes, a PPI network, and genes in BPs as input. It first maps genes in a BP into the PPI network to construct a BP-related subnetwork, which is expanded (in the whole PPI network) by a random walk with restart (RWR) process to generate a so-called expanded modularized network (eMN). Since the numbers of known disease genes are not high, an RWR process is also performed to generate an expanded disease-related gene list. For each eMN and disease, the expanded diabetes-related genes and disease-related genes are mapped onto the eMN. The association between diabetes and the disease is measured by the reachability of their genes on all eMNs, in which the reachability is estimated by a method similar to the Kolmogorov–Smirnov (KS) test. DIconnectivity_eDMN achieves an area under receiver operating characteristic curve (AUC) of 0.71 for predicting both Type 1 DRDs and Type 2 DRDs. In addition, DIconnectivity_eDMN reveals important BPs connecting diabetes and DRDs. For example, “respiratory system development” and “regulation of mRNA metabolic process” are critical in associating Type 1 diabetes (T1D) and many Type 1 DRDs. It is also found that the average proportion of diabetes-related genes interacting with DRDs is higher than that of non-DRDs.

## Introduction

With the increasing of human life-span, the incidence of diabetes is rapidly increasing, which presents a big threat to public health all over the world (Naslafkih and Sestier, 2003). According to a statistics from the International Diabetes Federation, approximately 415 million people worldwide suffered from diabetes in 2015, and the incidence is still increasing at a terrifying rate. By 2040, this number is estimated to exceed 640 million (International Diabetes Federation, 2015). Diabetes is a metabolic disease characterized by chronic hyperglycemia, which includes two forms, namely, Type 1 diabetes (T1D) and Type 2 diabetes (T2D). T2D accounts for about 85% of the diabetes incidences. Besides genetic factors, insulin resistance is a major risk factor for both T1D and T2D (Fourlanos et al., 2004). T1D and T2D also have a few common complications including damage to the kidneys, nerves, and cardiovascular systems, which may result in diabetes-related diseases (DRDs) like renal diseases (Papatheodorou et al., 2016, 2018). In general, DRDs can be divided into three categories: (1) microvascular disease, (2) macrovascular disease, and (3) miscellaneous complications. Microvascular disease mainly includes eye disease, kidney disease, and neuropathy; macrovascular disease mainly contains cardiovascular diseases; while miscellaneous complications include depression (Nouwen et al., 2011), dementia (Cukierman et al., 2005), and so on.

At present, people have explored the pathogenesis and pathological pathways of many DRDs. For example, inflammation, extracellular matrix expansion, oxidative stress, DNA damage, and vascular and nerve dysfunction are common pathways for the development of diabetic nephropathy (Wada and Makino, 2013; Jenkins et al., 2015; Zhang et al., 2018a); endothelial dysfunction and inflammation are involved in the development of diabetic vascular disease (Paneni et al., 2013); inflammation, endothelial dysfunction, and hypercoagulability are related to each other and play an important role in the occurrence of diabetic vascular disease (Domingueti et al., 2016). Though it is clear that certain biomarkers and biological pathways are involved in many DRDs, there is no systematic study summarizing DRD-associated common pathways, and pathways specific to the interaction between diabetes and specific DRDs.

With the development of high-throughput sequencing techniques, there are a lot of studies on genes and networks associated with diabetes and other diseases. For example, Ding et al. (2019) identified the core genes of T2D based on biological information, such as protein–protein interaction (PPI) network and microarray data. Zhang et al. (2018b) identified genes related to proliferative diabetic retinopathy based on PPI network and the random walk with restart (RWR) algorithm. Jiang et al. identified key genes and biological pathways related to diabetic nephropathy based on PPI network and microarray data (Jiang et al., 2015; Liu and Li, 2019; Song et al., 2019). The more and more accessible disease-related genes together with other important biological information, such as PPI data, gene expression data, and gene ontology (GO) data, provide us a unique opportunity for studying the interaction between diabetes and DRDs at the network level.

In this paper, we have proposed and compared 10 network-based computational methods to study the connections between diabetes and 254 diseases&vitamin D, which can generally be grouped into four categories, namely (1) DIcd based on the closest distance; (2) DIoverlap based on gene set overlap; (3) DINet based on random walk and gene set enrichment; (4) DIconnectivity based on cut edges between gene sets. Using these methods, we aim to predict DRDs, and perform a comprehensive analysis on important biological pathways associated with DRDs.

## Results

We have proposed four categories of algorithms to study the connections between diabetes and other diseases&vitamin D, namely, DIoverlap, DIcd, DINet, and DIconnectivity, all of which are based on PPI network/subnet. Since the diabetes-disease related genes might be enriched in a few biological processes (BPs) (Nigro et al., 2014), we also studied the connections based on BP modularized networks (MNs). The MN is constructed by mapping genes in each GO BP to the reference PPI network. In addition, we further expand each MN by an RWR procedure to construct the expanded MN (eMN). In our study, we set the expansion fold *N* to 3.

### An Overview of DIoverlap, DIcd, DINet, and DIconnectivity

**FIGURE 1:**
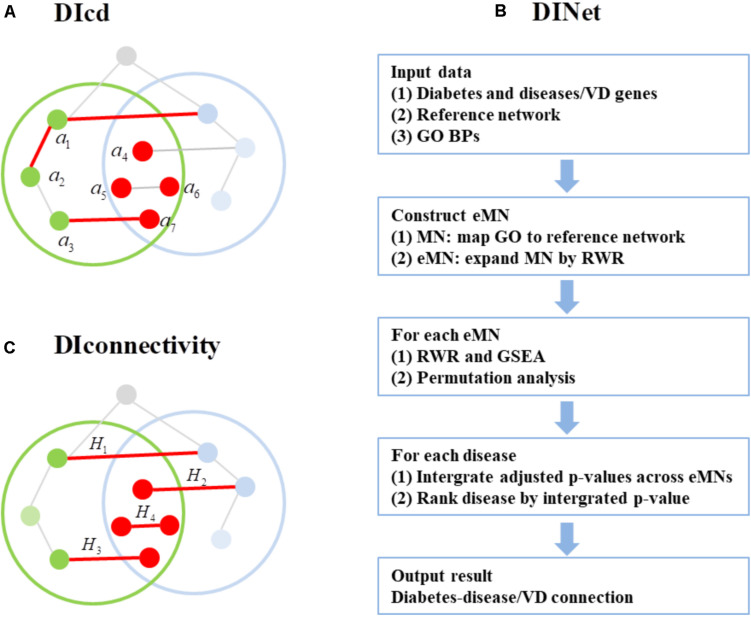
An overview of DIcd, DINet, andDIconnectivity. **(A)** The green dots and blue dots representthe genes of diabetes and disease, respectively, and the red dots represent their overlap. *a*_1_,*a*_2_,…,*a*_7_ represent diabetes genes, and the closest distances from *a*_*i*_(*i* = 1,2,…,7) to disease genes are 1, 2, 1, 0, 0, 0, 0, then DIcd = (1 + 2 + 1 + 0 + 0 + 0 + 0)/7 = 4/7. **(B)** An overview of GeroNet. RWR, random walk with restart; MN, modularized network; eMN, expanded modularized network; GSEA, gene set enrichment analysis. **(C)** The diabetes-disease pair is the same as **(A)**. Four kinds of interactions between the two gene sets were denoted by *H*_*1*_, *H*_*2*_, *H*_*3*_, *H*_*4*_. We assign the weight of the interactions between the overlap genes (*H*_*4*_) to 2, and the other types (*H*_*1*_, *H*_*2*_, *H*_*3*_) to 1. If the number of *H*_*i*_(*i* = 1,2,3,4) is *h*_*i*_, then DIconnectivity = *h*_1_ + *h*_2_ + *h*_3_ + 2*h*_4_.

DIoverlap is the Jaccard coefficient between diabetes and disease gene set. We applied this algorithm to three types of networks including the whole network, MN, and eMN, corresponding to DIoverlap-Whole network, DIoverlap-MN, and DIoverlap-eMN, respectively. We define the mean of Jaccard coefficients across the MNs/eMNs as the evaluation standard for DIoverlap-MN/DIoverlap-eMN. An overview of other three algorithms DIcd, DINet, and DIconnectivity is presented in Figure 1. For each algorithm, the disease-related genes were mapped to the network first. DIcd is the closest distance from diabetes genes to disease genes on PPI network (see Figure 1A). The major steps of DINet are shown in Figure 1B, which is similar to GeroNet algorithm (Yang et al., 2016). For DINet algorithm, the diabetes and disease genes were mapped to each eMN and the connection between the two mapped gene sets was estimated using RWR and gene set enrichment analysis (GSEA); the significance of the connection was evaluated by a permutation analysis, in which the diabetes genes are randomly permuted, and the significance *p*-value is adjusted for multiple testing; the connection between diabetes and disease/vitamin D is evaluated by the minimum adjusted *p*-value. The details of each step are presented in Section “Materials and Methods.”

DIconnectivity (Figure 1C) calculates the number of interactions between diabetes and disease gene set. We applied this algorithm to three types of networks including the whole network, MN, and eMN, corresponding to DIconnectivity-Whole network, DIconnectivity-MN, and DIconnectivity-eMN, respectively. We define the mean of interaction numbers across the MNs/eMNs as the evaluation standard for DIconnectivity-MN/DIconnectivity-eMN. In addition, DIconnectivity-eDMN calculates the interaction number between the expand diabetes and disease gene set on eMNs, and the gene sets are expanded by RWR and GSEA.

### Collection of Diabetes and Disease&vitamin D Genes, Reference PPI Network, GO BPs, and DRD Classification

We used diabetes/diseases genes collected from Enrichr as our input genes, and the genes of T1D/T2D/254 diseases were obtained by merging genes with the same human terms. Owing to some of the T1D/T2D/254 diseases also contain mouse or rat genes, we constructed two datasets: one of which only considers the human genes, called the H_Dataset, and the other one considers the genes of these three species, called HMR_Dataset. The vitamin D genes are obtained from GO terms which are related to vitamin D (i.e., the GO terms contain the word “vitamin D”) and the number of this gene set is 57. The number of disease genes in H_Dataset ranges from 298 to 3875 and a full list of disease&vitamin D genes is provided in Supplementary Dataset S1, while the number in HMR_Dataset ranges from 298 to 4134 and the gene list is provided in Supplementary Dataset S2. Besides, the number of T1D/T2D genes in H_Dataset is 355/2109, and the number is 2288/3521 in HMR_Dataset.

We used the PPI network compiled by Menche et al. as the reference network, and considered 3367 GO BPs to define MNs (see section “Materials and Methods”). We annotated the diseases&vitamin D as being either diabetes-related or non-diabetes related based on literature mining. 41 diseases&vitamin D were annotated as DRD1s (Supplementary Table S1) and 29 diseases&vitamin D were annotated as DRD2s (Supplementary Table S2).

### Comparison of DIoverlap, DIcd, DINet, and DIconnectivity

**FIGURE 2:**
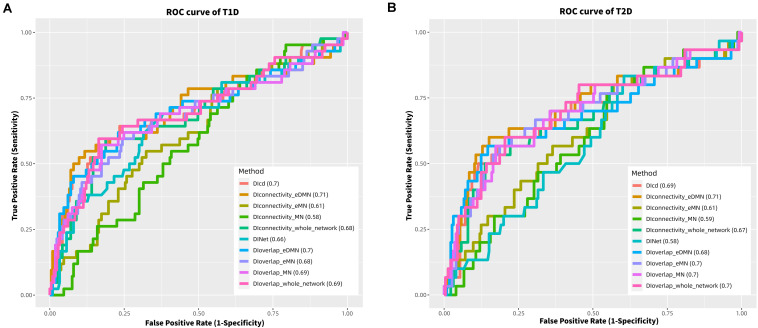
Comparison of different methods based on AUC of ROC in T1D **(A)** and T2D **(B)**. DIcd is based on whole PPI network; DINet is based on expanded modularized network (eMN); DIconnectivity_whole network represents DIconnectivity based on whole PPI network; DIconnectivity_MN represents DIconnectivity based on modularized network (MN); DIconnectivity_eMN represents DIconnectivity based on expanded modularized network (eMN); DIoverlap is defined similarly.

We used 10 methods to study the diabetes-disease&vitamin D connections based on PPI network/subnet, which are DIoverlap-Whole network, DIoverlap-MN, DIoverlap-eMN, DIoverlap-eDMN, DIcd, DINet, DIconnectivity-Whole network, DIconnectivity-MN, DIconnectivity-eMN, and DIconnectivity-eDMN. For DIoverlap-MN and DIconnectivity-MN, we only considered the MNs with the numbers of diabetes and disease/vitamin D mapping genes greater than 5, while for DIoverlap-eMN, DIconnectivity-eMN, DIoverlap-eDMN, and DIconnectivity-eDMN, we only considered the eMNs, which are expanded by these MNs. In addition, for DIoverlap-eDMN and DIconnectivity-eDMN, we also performed permutation training of eMNs (see Supplementary Material). For DINet, we considered the eMNs with the numbers of diabetes and disease/vitamin D mapping genes greater than 5. We compared the methods according to the accuracy of predicting the DRD1s/DRD2s. To quantify the performance, we calculated the area under the receiver operating characteristic curve (AUROC or simply AUC) for each method, a commonly used statistics to characterize the overall performance of a predictive model. For DINet, we tested nine values for parameter (i.e., 0.1, 0.2, …, 0.9) to get the best prediction result; for DIoverlap-eDMN/DIconnectivity-eDMN, we tested 10 values for expansion fold N (i.e., 1, 2, …, 10) on diabetes and diseases&vitamin D genes, and denoted the corresponding methods as DIoverlap-eDMN_EN/DIconnectivity-eDMN_EN. For T1D/T2D, DIconnectivity-eDMN_E3/DIconnectivity-eDMN_E4 performed the best with AUC of 0.71/0.71 on HMR_Dataset (Figure 2). In Figure 2, we only plotted the AUC result of each method under the optimal parameter (if parameter is included), and the parameter training results of different methods are listed in Supplementary Table S3.

### Diabetes Related Diseases Predicted by DIconnectivity-eDMN

We used the best performing method DIconnectivity_eDMN to predict the connections between diabetes and diseases&vitamin D, and the predicted ranking list of all 254 diseases&vitamin D related to T1D/T2D is provided in Supplementary Datasets S3, S4. It should be noted that we only considered the eMNs whose numbers of interactions between gene sets were greater than 0 for each diabetes-disease pair. In order to find significant related diseases, we converted the DIconnectivity into z-score statistics and calculated the *p*-values and then the diseases with *p*-values less than 0.05 were significant DRDs (Table 1). Finally, we found 22 significant related diseases of T1D/T2D. Among these DRDs, bacterial infection, acute myocardial infarction, atherosclerosis, osteoarthritis, and obesity are well-known DRDs. For bacterial infections, the mechanism of the susceptibility is the influence of glycemia on polymorphonuclear cell functions, such as urinary tract infection, “diabetic foot,” or “infectious cellulitis” (Schubert and Heesemann, 1995). Besides, certain infections (i.e., respiratory and foot infections) are overrepresented in the diabetic population and are associated with a higher risk of infection-related mortality (Pearson-Stuttard et al., 2016). On the one hand, diabetes increases the risk of acute myocardial infarction; on the other hand, acute myocardial infarction is the major cause of morbidity and mortality in diabetic patients (Echouffo-Tcheugui et al., 2018). The statistics from US centers for disease control and prevention (CDC^1^) also note that heart disease is the leading cause of death among people with diabetes. Diabetes is also associated with elevated odds of having osteoarthritis, which is the most frequent disease in individuals with diabetes (Rehling et al., 2019). The relationship between diabetes and obesity is more obvious (Weyer et al., 2001; Okada-Iwabu et al., 2013). According to the latest statistics from CDC, 89% of diabetes patients in the United States are overweight or obese (body mass index > 25 kg/m^2^). In Brazilian, 75% of the T2D patients are overweight, and 30% of them are obese (Gomes et al., 2006).

**TABLE 1:** The significant diabetes-related diseases inferred by DIconnectivity-eDMN.

**Disease**	***p*-value**	**DRD1**	**Disease**	***p*-value**	**DRD1**
**Type 1 diabetes**					
Systemic lupus erythematosus	1.24 E-04	1	Endometrial cancer	1.06 E-02	0
Breast cancer	4.45 E-04	0	Acute myocardial infarction	1.13 E-02	1
Bacterial infection	1.07 E-03	1	Endometriosis	1.58 E-02	0
Asthma	1.08 E-03	1	Cystic fibrosis	1.62 E-02	1
Ulcerative colitis	1.64 E-03	1	Huntington’s disease	2.29 E-02	0
Bipolar disorder	2.16 E-03	0	Multiple sclerosis	3.26E-02	1
Crown’s disease	2.49 E-03	1	Pancreatic cancer	3.58 E-02	1
Polycystic ovary syndrome	2.67 E-03	1	Osteoarthritis	4.14 E-02	0
Hypoxia	2.96 E-03	1	Obesity	4.31 E-02	1
Schizophrenia	3.77 E-03	0	Amyotrophic lateral sclerosis	4.36 E-02	0
Autism spectrum disorder	6.54E-03	0	Prostate cancer	4.84 E-02	0

**Disease**	***p*-value**	**DRD2**	**Disease**	***p*-value**	**DRD2**

**Type 2 diabetes**					
Systemic lupus erythematosus	6.52 E-04	0	Schizophrenia	1.10 E-02	0
Bacterial infection	7.74 E-04	1	Endometriosis	1.16 E-02	0
Asthma	1.72 E-03	0	Autism spectrum disorder	1.70 E-02	0
Breast cancer	1.73 E-03	1	Cystic fibrosis	1.76 E-02	0
Crown’s disease	2.13 E-03	1	Pancreatic cancer	2.66 E-02	1
Hypoxia	4.08E-03	1	Osteoarthritis	2.90 E-02	0
Bipolar disorder	5.09 E-03	0	Multiple sclerosis	3.07 E-02	0
Ulcerative colitis	5.64 E-03	0	Alzheimer’s disease	3.28 E-02	0
Polycystic ovary syndrome	6.99 E-03	1	Obesity	3.35E-02	1
Endometrial cancer	7.33 E-03	0	Huntington’s disease	4.76 E-02	0
Acute myocardial infarction	1.01 E-02	1	Atherosclerosis	4.95 E-02	1

It should be noted that for both T1D and T2D, systemic lupus erythematosus ranked first, which is associated with an increased risk of development of diabetes (Chung et al., 2007; Jiang et al., 2018). A cohort study in Toronto documented that women with SLE had a significantly higher prevalence of diabetes than the age-matched healthy controls (5 versus 1%) (Bruce et al., 2003). Therefore, we can conclude that SLE patients may develop diabetes. Followed in the list are breast cancer and asthma. According to Cancer Research UK^2^, women with diabetes have an increased risk of breast cancer. In addition, some studies have shown that diabetes not only increases the risk of breast cancer (Liao et al., 2011), but also increases the risk of breast cancer death (Luo et al., 2014; Bronsveld et al., 2015). The published data on disease occurrence showed that there was a strong positive association between T1D and asthma in Europe and elsewhere (Stene and Nafstad, 2001). Similarly, T2D has attracted attention as a risk factor for asthma (Murakami et al., 2019). Followed in the list are various types of psychiatric disorders, neurodegenerative diseases, and cancers. According to CDC, the complications of diabetes include heart disease, nerve damage, and mental health. On the other hand, some studies have shown that bipolar disorder (McIntyre et al., 2005), schizophrenia (Hoffman, 2017), and autism spectrum disorder (Alhowikan et al., 2019) also increase the prevalence of diabetes. In addition, high blood sugar can cause neuropathy (nerve damage) throughout your body, and some studies also suggested that there was an association between diabetes and the neurodegenerative diseases multiple sclerosis and amyotrophic lateral sclerosis (Mariosa et al., 2015; Tettey et al., 2015). Additionally, Cancer Research UK notes that people with diabetes have an increased risk of pancreatic cancer^3^. What is more, several studies show a higher risk of womb cancer in women with diabetes^4^. We should also note that diabetes is one of the common comorbidities of ulcerative colitis (Maconi et al., 2014) and cystic fibrosis (Prentice et al., 2016; Hart et al., 2018).

DIconnectivity-eDMN can effectively rank some recognized DRDs at the top of the list, but there are still some obvious related diseases that are relatively backward, such as diabetic nephropathy of T1D (48th), insulin resistance of T2D (68th), and even put some diabetes related diseases at the bottom of the list, such as vitamin D (255th) and morbid obesity (240th) of T1D/T2D. Such a ranking error may be due to incomplete genes in our network or diseases&vitamin D. In addition, some DRDs were not defined as DRDs, but we did find evidence to support their connections, such as bipolar disorder, endometrial cancer, and osteoarthritis.

### Functional Subnets Connecting Diabetes and Diseases&vitamin D

For each diabetes-disease/vitamin D connection, we consider eMNs satisfying the following two conditions: (1) the eMNs are under the optimal permutation result; (2) the interaction numbers between diabetes and disease/vitamin D mapping genes in the eMNs are greater than 0. In the 255 T1D/T2D-disease&vitamin D connections, the number of eMNs ranges from 295/298 to 3123/3291, total 427,349/431,778 eMNs. Generally speaking, not all subnets play an important role in the diabetes-disease&vitamin D connections, so we identify the significant eMNs for each diabetes-disease/vitamin D connection with permutation analysis method, and the specific steps are as follows: (1) permute diabetes genes in each eMN for 100 times to calculate the null distribution of DIconnectivity with DIconnectivity-eDMN_E3 for T1D and DIconnectivity-eDMN_E4 for T2D and (2) convert the DIconnectivity to a z-score statistic based on this null distribution, then a *p*-value is estimated and adjusted for multiple testing. We consider the eMNs with FDR ≤ 0.05 are significant, and the number of significant eMNs for T1D/T2D-disease&vitamin D connections ranges from 46/0 to 1908/1284, a total of 214,545 (∼50.2%)/84,165 (∼19.5%) significant eMNs.

### Functional Subnets Connecting T1D and Diseases&vitamin D

**FIGURE 3:**
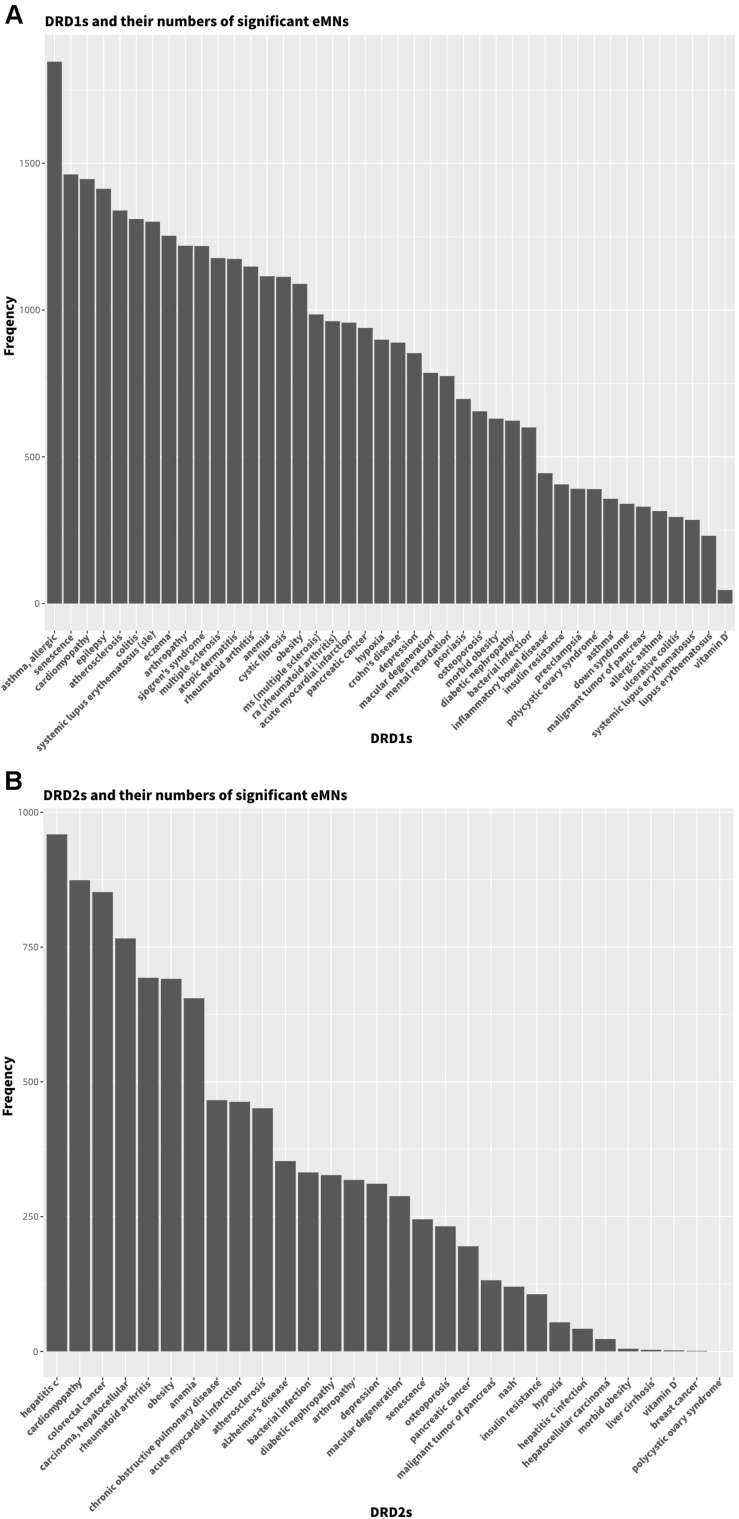
The numbers of significant eMNs in T1D-DRD1 connections **(A)** and T2D-DRD2 connections **(B)**.

It is worth noting that different eMNs have different frequencies to connect diabetes and diseases&vitamin D, that is, some eMNs are involved in multiple diabetes-disease&vitamin D connections, and some only affect a few or specific ones. In order to study eMN frequency in the T1D-disease&vitamin D connections, we calculated the frequencies of all significant eMNs for each connection, and the average frequency (AF) was used as its eMN frequency. Among 255 T1D-disease&vitamin D (42 DRD1s and 213 non-DRD1s) connections (AF∈[102, 201]), there are 92 connections with AF less than 150, of which 23 are DRD1s involved and 69 are non-DRD1s involved. This shows that 55% of T1D-DRD1 connections have an eMN frequency of less than 150, while for non-DRD1s, this proportion is only 32%. Obviously, the smaller the eMN frequency, the higher the specificity, and then we can conclude that DRD1s have higher eMN specificity to connect T1D compared to non-DRD1s. The AFs of 42 connections (DRD1s involved) are plotted in Figure 3A, and from the figure, we can see that some well-known DRD1s have low frequencies (e.g., morbid obesity and diabetic nephropathy). The higher frequent diseases include heart diseases (e.g., cardiomyopathy and atherosclerosis) and inflammatory diseases (e.g., colitis and eczema), which suggests that the connections between T1D and DRD1s may be mediated by eMNs with very different frequencies.

In order to further search for specific eMNs and non-specific eMNs of T1D-DRD1 connections, we defined the specific index SP (SP = KF/AF, 0 < KF≤42, 0 < AF≤255, 0 < SP≤1), where KF is the frequency of significant eMN in the range of T1D-DRD1 connections. It is easy to know that when AF is closer to KF and KF is closer to 42, the eMN specificity is higher. Therefore, we set the SP threshold to 0.3 (Supplementary Table S4), i.e., the eMN with SP greater than 0.3 is defined as specific eMN, otherwise non-specific eMN. We sorted the specific eMNs according to KF from large to small, and list the top 20 non-specific eMNs and specific eMNs in Table 2. Non-specific eMNs include BPs such as “GO:0060070_canonical Wnt signaling pathway” and “GO:0060828_regulation of canonical Wnt signaling pathway.” According to Table 2, there are 41 DRD1s (42 in total) and 196 non-DRD1s (213 in total) that are significantly related to the eMN “GO:0060070_canonical Wnt signaling pathway,” and there are 41 (98%) DRD1s and 185 (87%) non-DRD1s that are significantly related to the eMN “GO:0060828_regulation of canonical Wnt signaling pathway.” The Wnt signaling pathway has been reported to be associated with glucose and lipid metabolism (Qin et al., 2018). Besides, many studies have shown that the Wnt signaling pathway is related to the pathogenesis of diabetic nephropathy (Kavanagh et al., 2011) and diabetic retinopathy (Chen and Ma, 2017). In the non-specific eMNs, except for multiple pathways related to Wnt signaling (GO:0016055_Wnt signaling pathway, GO:0198738_cell-cell signaling by wnt, GO:0030111_regulation of Wnt signaling pathway), there are also eMNs related to kidney development, such as GO:0072001_renal system development, GO:0001822_kidney development, and GO:0001655_urogenital system development.

**TABLE 2:** Top 20 non-specific and specific eMNs (KF from large to small) of T1D-DRD1connections.

**Non-specific eMNs**	**KF**	**AF**	**SP**
GO:0060070_canonical Wnt signaling pathway	41	237	0.172995781
GO:0060828_regulation of canonical Wnt signaling pathway	41	226	0.181415929
GO:2000027_regulation of animal organ morphogenesis	41	224	0.183035714
GO:0000226_microtubule cytoskeleton organization	40	244	0.163934426
GO:0022604_regulation of cell morphogenesis	40	240	0.166666667
GO:0051090_regulation of DNA-binding transcription factor activity	40	243	0.164609053
GO:0050769_positive regulation of neurogenesis	40	243	0.164609053
GO:0051047_positive regulation of secretion	40	243	0.164609053
GO:0042391_regulation of membrane potential	40	249	0.16064257
GO:0002793_positive regulation of peptide secretion	40	233	0.17167382
GO:0016055_Wnt signaling pathway	39	241	0.161825726
GO:0198738_cell-cell signaling by wnt	39	240	0.1625
GO:0030111_regulation of Wnt signaling pathway	39	226	0.172566372
GO:0016050_vesicle organization	39	224	0.174107143
GO:0022412_cellular process involved in reproduction in multicellular organism	39	229	0.170305677
GO:0050804_modulation of chemical synaptic transmission	39	240	0.1625
GO:0051091_positive regulation of DNA-binding transcription factor activity	39	235	0.165957447
GO:0072001_renal system development	39	243	0.160493827
GO:0001822_kidney development	39	234	0.166666667
GO:0001655_urogenital system development	39	222	0.175675676
**Specific eMNs**			
GO:0060541_respiratory system development	28	91	0.307692308
GO:1903311_regulation of mRNA metabolic process	23	75	0.306666667
GO:1902105_regulation of leukocyte differentiation	20	59	0.338983051
GO:0052548_regulation of endopeptidase activity	20	66	0.303030303
GO:0007517_muscle organ development	18	53	0.339622642
GO:0071383_cellular response to steroid hormone stimulus	18	47	0.382978723
GO:0031100_animal organ regeneration	18	56	0.321428571
GO:0060537_muscle tissue development	17	49	0.346938776
GO:0009267_cellular response to starvation	17	48	0.354166667
GO:0003007_heart morphogenesis	17	51	0.333333333
GO:0021782_glial cell development	16	52	0.307692308
GO:0048545_response to steroid hormone	16	53	0.301886792
GO:0002521_leukocyte differentiation	16	42	0.380952381
GO:0071901_negative regulation of protein serine/threonine kinase activity	16	42	0.380952381
GO:0048771_tissue remodeling	16	46	0.347826087
GO:0042110_T cell activation	16	52	0.307692308
GO:0048732_gland development	16	51	0.31372549
GO:0043434_response to peptide hormone	15	46	0.326086957
GO:0051169_nuclear transport	15	42	0.357142857
GO:0036473_cell death in response to oxidative stress	15	44	0.340909091

Specific eMNs include BPs such as “respiratory system development” and “regulation of mRNA metabolic process.” There are 28 (68%) DRD1s and 63 (30%) non-DRD1s that are significantly related to “respiratory system development,” and there are 23 (55%) DRD1s and 52 (24%) non-DRD1s that are significantly related to “regulation of mRNA metabolic process.” Related studies have shown that respiratory control imbalance is common in T1D patients (Bianchi et al., 2017). The available evidence shows that diabetes usually changes metabolites such as glucose, fructose, amino acids, and lipids through metabolic pathways (Arneth et al., 2019). In addition, the well-known specific eMNs of diabetes, insulin related BPs (GO:0032868_response to insulin, GO:0032869_cellular response to insulin stimulus) are also in the list (Brezar et al., 2011).

### Functional Subnets Connecting T2D and Diseases&vitamin D

We conducted a similar analysis for T2D. Among 255 T2D-diseases&vitamin D (30 DRD2s and 225 non-DRD2s) connections (AF∈[10, 209]), there are 94 connections with AF less than 100, of which 16 are DRD2s involved and 78 are non-DRD2s involved. This shows that 53% of T2D-DRD2 connections have an eMN frequency of less than 100, while for non-DRD2s, this proportion is only 35%. The AFs of 30 connections (DRD2s involved) are plotted in Figure 3B, and from the figure, we can see that high frequent diseases include obesity and some heart diseases (cardiomyopathy, acute myocardial infarction, and atherosclerosis).

We set the SP threshold to 0.2 (Supplementary Table S5) to define specific eMNs and non-specific eMNs, and list the top 20 of them in Table 3. The non-specific eMN with the largest KF is “GO:0000226_microtubule cytoskeleton organization,” and there are 22 DRD2s (30 in total) and 178 non-DRD2s (225 in total) that are significantly related to it. Studies have found that microtubule polymerization may play an important role in glucose transport (Taneja and Priyadarshini, 2018). It is worth noting that pathways related to Wnt signaling are also significantly related to T2D, such as GO:0198738_cell-cell signaling by wnt, GO:0016055_Wnt signaling pathway, GO:0030111_regulation of Wnt signaling pathway, and GO:0060828_regulation of canonical Wnt signaling pathway, and there are evidences that the Wnt signaling pathway is a key pathway for the occurrence of T2D (Lee et al., 2008; Liu et al., 2018). Therefore, we can conclude that both T1D and T2D are significantly related to the Wnt signaling pathway. On the other hand, the Wnt signaling pathway is also related to the development of some DRD2s, for example, miR-128-3p aggravates cardiovascular calcification and insulin resistance in T2D rats by downregulating ISL1 through the activation of the Wnt pathway (Wang et al., 2019). The specific eMN with the largest KF is “GO:0061138_morphogenesis of a branching epithelium,” and studies have found that branching morphogenesis is a critical step in the development of many epithelial organs, for example, lung (Carter et al., 2014; Goodwin et al., 2019), kidney (Basson et al., 2006), and breast, besides, breast epithelial branch morphogenesis may be related to breast cancer (Kessenbrock et al., 2017). In addition, the similar BPs of morphogenesis of a branching epithelium (GO:0060562_epithelial tube morphogenesis ranked 6, GO:0001763_morphogenesis of a branching structure ranked 22 and GO:0048754_branching morphogenesis of an epithelial tube ranked 25) are also in the specific eMN list, which further indicates that the BP of morphogenesis of a branching epithelium structure is important for T2D-DRD2 connections.

**TABLE 3:** Top 20 non-specific and specific eMNs (KF from large to small) of T2D-DRD2 connections.

Non-specific eMNs	**KF**	**AF**	**SP**
GO:0000226_microtubule cytoskeleton organization	22	200	0.11
GO:0051052_regulation of DNA metabolic process	22	206	0.106796117
GO:0016570_histone modification	22	184	0.119565217
GO:0198738_cell-cell signaling by wnt	22	182	0.120879121
GO:0016055_Wnt signaling pathway	22	178	0.123595506
GO:0090068_positive regulation of cell cycle process	22	191	0.115183246
GO:0048285_organelle fission	22	209	0.105263158
GO:0045787_positive regulation of cell cycle	22	195	0.112820513
GO:0045930_negative regulation of mitotic cell cycle	22	187	0.117647059
GO:0034660_ncRNA metabolic process	21	176	0.119318182
GO:0051260_protein homooligomerization	21	165	0.127272727
GO:0000082_G1/S transition of mitotic cell cycle	21	180	0.116666667
GO:0072331_signal transduction by p53 class mediator	21	176	0.119318182
GO:1901987_regulation of cell cycle phase transition	21	196	0.107142857
GO:0031396_regulation of protein ubiquitination	21	167	0.125748503
GO:1901990_regulation of mitotic cell cycle phase transition	21	189	0.111111111
GO:0060249_anatomical structure homeostasis	20	202	0.099009901
GO:0016569_covalent chromatin modification	20	144	0.138888889
GO:0060070_canonical Wnt signaling pathway	20	172	0.11627907
GO:0044843_cell cycle G1/S phase transition	20	166	0.120481928
**Specific eMNs**			
GO:0061138_morphogenesis of a branching epithelium	14	67	0.208955224
GO:0007626_locomotory behavior	13	60	0.216666667
GO:0001890_placenta development	13	65	0.2
GO:0007162_negative regulation of cell adhesion	12	55	0.218181818
GO:0001894_tissue homeostasis	12	50	0.24
GO:0060562_epithelial tube morphogenesis	12	43	0.279069767
GO:0034101_erythrocyte homeostasis	12	55	0.218181818
GO:0048469_cell maturation	12	52	0.230769231
GO:0009267_cellular response to starvation	11	45	0.244444444
GO:0007179_transforming growth factor beta receptor signaling pathway	11	44	0.25
GO:0042594_response to starvation	11	43	0.255813953
GO:0048762_mesenchymal cell differentiation	11	42	0.261904762
GO:0051100_negative regulation of binding	11	53	0.20754717
GO:0051047_positive regulation of secretion	11	39	0.282051282
GO:0030098_lymphocyte differentiation	11	50	0.22
GO:0001558_regulation of cell growth	11	46	0.239130435
GO:0006732_coenzyme metabolic process	11	42	0.261904762
GO:0032259_methylation	10	41	0.243902439
GO:0090287_regulation of cellular response to growth factor stimulus	10	46	0.217391304
GO:0019359_nicotinamide nucleotide biosynthetic process	10	41	0.243902439

### Key Connectors Mediating Diabetes-Disease Connections in Significant Subnets

**FIGURE 4:**
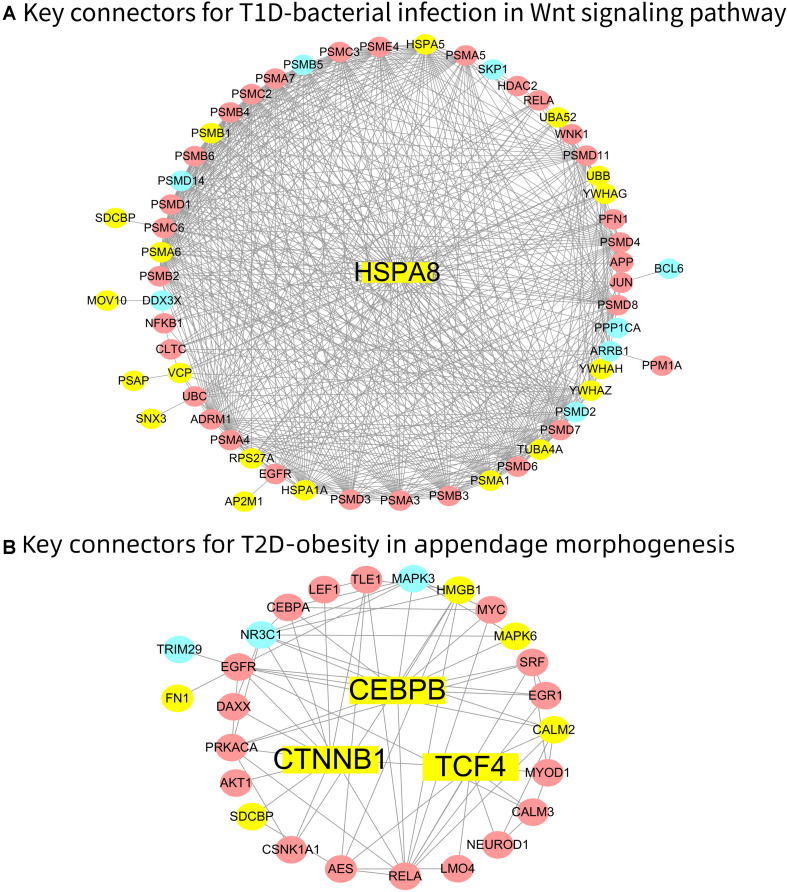
Key connectors of **(A)** T1D-bacterial infection in Wnt signaling pathway and **(B)** T2D-obesity in appendage morphogenesis. We use node shape to denote key connectors: (1) square represents the key connectors; (2) circle represents diabetes and disease genes. We use fill color to denote diabetes and disease information: (1) red represents diabetes gene; (2) blue represents disease gene; and (3) yellow represents the overlapping diabetes and disease gene.

We performed key connector analysis (KCA) to infer key genes that connect diabetes and DRDs in selected eMNs. The detailed information of KCA is provided in Section “Materials and Methods.” We selected two common diabetes-disease connections including T1D-bacterial infection and T2D-obesity as case studies to illustrate the key connectors (Figure 4). In Figure 4, we only show the subnet consisting of key connectors and their neighboring genes for a better view.

The T1D-bacterial infection connection is most significant in the eMN corresponding to “GO:0016055_Wnt signaling pathway.” In this eMN, there are 111 T1D genes and 58 bacterial infection genes, and the number of overlap between them is 30. We analyzed these 30 common genes with key driver analysis (KDA), and the key connector gene HSPA8 was obtained (Figure 4A). Studies have shown that HSPA8 binds bacterial lipopolysaccharide (LPS) and mediates LPS-induced inflammatory response (Yahata et al., 2000; Triantafilou et al., 2001). Similarly, T2D-obesity connection is the most significant in the eMN corresponding to “GO:0035107_appendage morphogenesis.” In this eMN, there are 84 T2D genes and 30 obesity genes, and the number of overlap between them is 23. We analyzed these 23 common genes, and got the key connector genes TCF4, CTNNB1 and CEBPB (Figure 4B). TCF4 (TCF7L2) is the strongest T2D candidate gene discovered to date, and it also plays a key role in the development and function of adipose tissue (Chen et al., 2018). CTNNB1 (β-catenin) is a key regulator of fat expansion and human obesity (Chen et al., 2020). Besides, studies have shown that co-administration of insulin and leptin to pancreatic islet-derived mesenchymal stem cell (PID-MSC) leads to the co-development of insulin and leptin resistance, and the differentiation signaling is mainly mediated by CTNNB1 and Tub (Ercin et al., 2018). In addition, the repressed expression of CEBPB has been found in obesity and T2D in adipose tissue (Li et al., 2016).

## Discussion

### The Effect of the Number of Disease Genes on the Result

In the predicted diabetes-related diseases&vitamin D list, some well-known diseases are not ranked at the top (e.g., diabetic nephropathy, insulin resistance) and we speculate that it may be caused by the difference in the number of known disease genes. We sorted the numbers of diseases&vitamin D genes from large to small, and found that diabetic nephropathy ranked 118 and vitamin D ranked 255. In order to prove our conjecture, we expanded (limited) the genes of each disease to 500 and predicted DRDs with DIconnectivity_eDMN method on HMR_Dataset (Supplementary Table S6). We found that the predicted top five related diseases of T1D and T2D are the same, all of which are “essential thrombocytemia,” “bacterial infection,” “osteoporosis,” “diabetic nephropathy,” “insulin resistance.” This result shows that the number of known disease genes indeed affects the ranking of related diseases to a certain extent. Additionally, vitamin D has the least number of genes and is predicted at the bottom of the list, which further proves our conjecture.

### Diabetes Genes Play an Important Role in the Diabetes-Disease Connections

We calculated the proportion of T1D genes involved in the DIconnectivity (DIconnectivity-Whole network) for each T1D-DRD1 connection, and found that the average proportion was 0.64, while in T1D-non-DRD1 connections, the proportion was only 0.54. Similarly, in T2D-DRD2 connections and T2D-non-DRD2 connections, the average proportions were 0.59 and 0.51, respectively. In addition, we also calculated the proportion of disease genes involved in the corresponding DIconnectivity, and found that the average proportions of DRD1s and non-DRD1s were 0.80 and the average proportions of DRD2s and non-DRD2s were 0.88 and 0.87, respectively. The average proportion of disease genes is higher than that of diabetes, but the proportions is the same for DRDs and non-DRDs, which shows that diabetes plays a key role in diabetes-disease connections.

For the shortest path method, we considered three distance measures (Guney et al., 2016): (1) the shortest distance *d*_*s*_(*A*,*S*), $d_{s}\,\left(A,S\right)=\frac{1}{||S||}\,\sum_{a\in A}\frac{1}{||A||}\,\sum_{s\in S}d\,\left(a,s\right)$, where *A* is diabetes gene set, *S* is disease gene set, and *d*(*a*,*s*) is the shortest path length between nodes *a* and *s* in PPI network; (2) the closest distance *d*_*c*_(*S*,*A*), $d_{c}\,\left(S,A\right)=\frac{1}{||S||}\,\sum_{s\in S}\min_{a\in A}d\,\left(s,a\right)$, *d*(*s*,*a*) = *d*(*a*,*s*); (3) the closest distance *d*_*c*_(*A*,*S*), $d_{c}\,\left(A,S\right)=\frac{1}{||A||}\,\sum_{a\in A}\min_{s\in S}d\,\left(a,s\right)$. We found that *d*_*c*_(*A*,*S*) has the best results (Supplementary Table S7). Among these three methods, *d*_*s*_(*A*,*S*) considers all genes of diabetes and disease, *d*_*c*_(*S*,*A*) only considers all genes of disease, and *d*_*c*_(*A*,*S*) only considers all genes of diabetes. Therefore, we can conclude that diabetes plays a more important role in diabetes-disease connections.

### The Important Genes and Distances in the Diabetes-Disease Connections

DIoverlap method takes the intersection between diabetes and disease gene sets as a criterion for measuring their connection. In essence, it only considers the genes with distances of 0; DIconnectivity method considers the genes with distances of 0 and 1; DIcd method considers all diabetes genes regardless of distance. Among the three methods, DIconnectivity_eDMN performs best, which shows that the genes with distances of 0 and 1 play an important role in the diabetes-disease connections.

### The Impact of BP Redundancy

In order to evaluate the impact of BP redundancy on prediction results, we calculated the semantic similarity among 3367 BP terms using R software package GOSemSim, of which 1141/359/59 terms have semantic similarity less than 0.8/0.7/0.6. Too high similarity and few terms are not our selection criteria, so we adopted the optimal method DIconnectivity-eDMN to predict DRDs again based on eMNs with similarity less than 0.7. Through the training of DIconnectivity-eDMN_EN (N = 1, 2,…, 10), we found that DIconnectivity-eDMN_E3/DIconnectivity-eDMN_E4 has the best prediction for DRD1s/DRD2s with AUC of 0.70/0.71. Therefore, we can conclude that removing a few highly similar terms has very little impact on the prediction effect.

## Materials and Methods

### Database

We downloaded the upregulated and downregulated gene files of diabetes/diseases Disease_Perturbations_ from_GEO_up.txt (Supplementary Dataset S5) and Disease_ Perturbations_ from_GEO_down.txt (Supplementary Dataset S6) from Enrichr^5^. Enrichr is a comprehensive resource for curated gene sets, currently containing 180,184 annotated gene sets from 102 gene set libraries (Kuleshov et al., 2016). Terms of these two files are the same, but the corresponding genes are different, so we first merge the upregulated and downregulated genes of each term, and get a total of 839 terms of human, mouse, and rat. In addition, since some diabetes/diseases terms are the same but only the case of the first letter is different, so we merged the same human terms of diabetes/diseases. Finally, we obtained a list of genes for 254 diseases and T1D/T2D (Supplementary Dataset S1). Besides, we also extracted vitamin D genes from GO terms which were related to vitamin D. In addition, we found that diabetes and some of 254 diseases not only contain human term genes, but also mouse or rat term genes, so we constructed another dataset by adding them to the corresponding disease gene set (Supplementary Dataset S2).

We used the human PPI network compiled by Menche et al. as the reference PPI network (Guney et al., 2016), and conducted research based on its largest connected subnet, which consists of 13,329 proteins and 141,150 protein interactions.

Gene ontology terms were obtained based on R software package GO.db. We consider GO BPs containing 30–500 genes, and ignore either very small or overly large functional gene sets. Finally, we obtained 3367 GO BPs to generate various network modules.

### Diabetes–Disease/Vitamin D Connection Annotation

We adopted literature mining approach to annotate whether a disease/vitamin D is diabetes-related. Specifically, we ranked diseases&vitamin D based on their Jaccard indices between their names and the term “type 1 diabetes” (“type 2 diabetes”) in PubMed abstracts published from 2008 to 2019. The PubMed abstracts containing the term “type 1 diabetes” (“type 2 diabetes”) from 2008 to 2019 were retrieved using Entrez Programming Utilities^6^. The term “type 1 diabetes” corresponds to “type 1 diabetes” [MeSH Terms] OR “type 1 diabetes” [All Fields] in PubMed, which is a superset of the term “type 1 diabetes.” The co-occurrence of disease and diabetes was evaluated by the following equation:

$$J\,\text{a}\,c\,c\,a\,r\,d\,\left(d\,i\,s\,e\,a\,s\,e,d\,i\,a\,b\,e\,t\,e\,s\right)=\frac{\left|P\,u\,b\,M\,e\,d\,I\,D_{d\,i\,s\,e\,a\,s\,e}\cap P\,u\,b\,M\,e\,d\,I\,D_{d\,i\,a\,b\,e\,t\,e\,s}\right|}{\left|P\,u\,b\,M\,e\,d\,I\,D_{d\,i\,s\,e\,a\,s\,e}\cup P\,u\,b\,M\,e\,d\,I\,D_{d\,i\,a\,b\,e\,t\,e\,s}\right|}$$

Where *PubMedID*_*disease*_ and *PubMedID*_*diabetes*_ were the PubMed IDs containing the disease name and the term “diabetes,” respectively.

According to previous study, some diseases are indeed associated with diabetes, such as diabetic nephropathy, obesity, and bacterial infection (Forbes and Cooper, 2013). We used the minimum Jaccard coefficient of these diseases as the threshold, and selected the diseases&vitamin D with Jaccard coefficient larger than threshold as DRDs. Finally, we obtained 41 diseases&vitamin D that are defined as DRD1s and 29 diseases&vitamin D that are defined as DRD2s.

### Four Categories of Algorithms

We used four algorithms to identify the connections between diabetes and diseases&vitamin D, namely DIoverlap rank the diseases&vitamin D by calculating the Jaccard coefficient, DIcd performed by using the closest distance. DINet based on a procedure similar to gene set enrichment analysis and an RWR procedure, and DIconnectivity based on the number of interactions between diabetes and diseases&vitamin D genes.

#### DIcd

DIcd is a closest distance method: let *A* and *S* denote diabetes and disease gene set, respectively, and *d*_*c*_(*A*,*S*) is the closest distance from *A* to *S*. Given two nodes *a* ∈ *A* and *s* ∈ *S*, the shortest path length between *a* and *s* in the network is represented by *d*(*a*,*s*), then we define *d*_*c*_(*A*,*S*) as follows:

$$d_{c}\,\left(A,S\right)=\frac{1}{||A||}\,\sum_{a\in A}\min_{s\in S}d\,\left(a,s\right)$$

It should be noted that the smaller the value of DIcd, the higher the connection between diabetes and disease.

#### DIoverlap

DIoverlap is the Jaccard coefficient between diabetes and disease gene set, and the larger the value, the higher the connection between them.

#### DINet

DINet is similar to the GeroNet (Yang et al., 2016, 2017) and it consists of three steps: (1) Generate expanded network modules (eMN), (2) Calculate the enrichment scores on eMNs follow a method similar to GSEA, and (3) Calculate the significance of enrichment score based on permutation test.

Step 1: To generate expanded network modules (eMN), we map each GO BP to the reference PPI network to generate the corresponding MN, which is further expanded by an RWR (see Supplementary Material) until it reaches *N* times the original gene size and the maximum does not exceed 500 genes.

Step 2: To calculate the diabetes-disease enrichment score on an eMN, we first map the two gene sets to the eMN and perform two RWR expansions by setting the two mapped gene sets as seeds, which will rank all genes in the eMN, respectively. We go through the sorted gene list of eMN based on disease (diabetes) gene seed, if we encounter a gene that is not a diabetes (disease) gene, $-\sqrt{\frac{G}{N-G}}$ is added to the score, where *N* is the number of genes for the network, and *G* is the number of diabetes (disease) genes; otherwise, $\sqrt{\frac{N-G}{G}}$ is added. This generates a curve and the peak value is defined as *ES*_*1*_(*ES*_*2*_). The enrichment score is defined as the weighted sum of scores

$$E\,S_{\beta }=\beta \,E\,S_{1}+\left(1-\beta \right)\,E\,S_{2},0<\beta <1.$$

Step 3: To calculate the significance of enrichment score, we permute diabetes genes in the eMN for 100 times to calculate the null distribution of enrichment scores and convert the *ES*_β_ to a z-score statistic based on this null distribution, then a *p*-value is estimated and adjusted for multiple testing. For each diabetes-disease connection, the significance is defined as the minimum adjusted *p*-value of eMN. The diseases are then ranked based on their significances, and the more significant the disease, the more diabetes-related.

#### DIconnectivity

DIconnectivity is the weighted sum of interaction numbers between diabetes and disease gene set, which is based on the idea of cut edge. We can divide the interactions between the two gene sets into four categories: (1)*H*_*1*_: one gene involved in the interaction is disease/VD gene and the other gene is diabetes gene; (2)*H*_*2*_: one gene is disease/VD gene, and the other is an overlap gene (both a disease gene and a diabetes gene); (3)*H*_*3*_: One gene is a diabetes gene, and the other is an overlap gene; (4) *H*_*4*_: the two genes are both overlap genes. We give the weight of the number of *H*_*i*_(*i* = 1,2,3) as 1, and the weight of *H*_*4*_ as 2 (see Figure 1C). In addition, we also proposed DIconnectivity-eDMN method, which calculates the weighted sum of interaction numbers between the expand diabetes and disease gene set. The gene sets are expanded based on RWR and GSEA: (1) In Step 2 of DINet, we can obtain the score of each diabetes/disease gene; (2) Sort the diabetes/disease genes in descending order according to their scores; (3) The top *n* genes are defined as expanded diabetes/disease genes (Hu et al., 2018), and *n* is *N* times the original gene size and the maximum does not exceed the number of eMN genes. For each diabetes-disease pair, its DIconnectivity is defined as the mean of interaction numbers across eMNs. The larger the value is, the higher the connection between them.

#### Key Connector Analysis

We adopted the KDA software package (Zhang and Zhu, 2013) to identify key connectors in PPI network. KDA was originally designed to identify “key regulators” in a directed regulatory network. When applied to undirected networks like PPI networks, we consider the key nodes as “key connectors” since they do not necessarily contain the directional information (Zhang and Zhu, 2013). Such key connectors function more like a “hub” gene, instead of being considered as “master regulators.” Specifically, KDA takes a set of genes *G* and an undirected gene network *N* as inputs. It has two searching strategies, namely, dynamic neighborhood search (DNS) and static neighborhood search (SNS) for identifying key connectors. We adopted DNS in this study: (1) It first generates a subnet *N*_*G*_ consisting of all nodes in *N* with no more than *L*(*L=2* in this study) steps away from the nodes in *G*. (2) For each gene *g* in *N*_*G*_, DNS then searches for genes with distances no more than *h* = 1,2,…,*H*(*H=2* in this study) in *N*_*G*_. The set of genes (not including *g*) is denoted by *N*_*G*_(*HLN*_*g*,*h*_). The hypergeometric test is then used to calculate the enrichment between *N*_*G*_(*HLN*_*g*,*h*_) and *G* with the genes in *N*_*G*_ as background for each *h*. The final enrichment *p*-value of each gene *g* is calculated as the minimum *p*-value across *h* layers. (3) The Bonferroni correction is performed to adjust for multiple testing and the genes with significant Bonferroni *p*-values (≤0.05) are outputted as key connectors.

## Data Availability Statement

The original contributions presented in the study are included in the article/Supplementary Material. Further inquiries can be directed to the corresponding author/s.

## Author Contributions

SC, HZ, and LZ conceived the concept of the work. LZ, JX, QW, AW, CL, GT, and HZ performed the experiments. LZ wrote the manuscript. All authors approved the final version of this manuscript.

## Conflict of Interest

AW, CL, and GT were employed by Geneis Beijing Co., Ltd. The remaining authors declare that the research was conducted in the absence of any commercial or financial relationships that could be construed as a potential conflict of interest.

## Footnotes

1 https://www.cdc.gov/diabetes/managing/problems.html

2 https://www.cancerresearchuk.org/about-cancer/breast-cancer/risks-causes/risk-factors

3 https://www.cancerresearchuk.org/about-cancer/pancreatic-cancer/risks-causes

4 https://www.cancerresearchuk.org/about-cancer/womb-cancer/risks-causes

5 https://maayanlab.cloud/Enrichr/

6 http://eutils.ncbi.nlm.nih.gov/entrez/eutils/esearch.fcgi?db~=~pubmed&term~=~[type 1 diabetes]+AND+2008:2019 [pdat]&retmax~=~999999

## Supplementary Material

The Supplementary Material for this article can be found online at: https://www.frontiersin.org/articles/10.3389/fgene.2020.617136/full#supplementary-materialDIconnectivity-eDMN is available at https://github.com/zhulijuan123/connectivity.
