# Supplementary material for: Revealing the Interactions Between Diabetes, Diabetes-Related Diseases, and Cancers Based on the Network Connectivity of Their Related Genes
Source: Front Genet. 2020 Dec 14;11:617136. doi: 10.3389/fgene.2020.617136 (PMC7767993; doi:10.3389/fgene.2020.617136)
Supplement: Supplementary file 2 [file Data_Sheet_2.docx]

Supplementary Material

# Supplementary Tables

Table S1. The DRD1s based on literature mining.

| **Rank** | **DRD1s** | **Rank** | **DRD1s** |
| --- | --- | --- | --- |
| **1** | diabetic nephropathy | **22** | ra (rheumatoid arthritis) |
| **2** | insulin resistance | **23** | anemia |
| **3** | rheumatoid arthritis | **24** | sjogren's syndrome |
| **4** | vitamin D | **25** | ms (multiple sclerosis) |
| **5** | obesity | **26** | cystic fibrosis |
| **6** | multiple sclerosis | **27** | colitis |
| **7** | lupus erythematosus | **28** | senescence |
| **8** | systemic lupus erythematosus | **29** | hypoxia |
| **9** | atherosclerosis | **30** | ulcerative colitis |
| **10** | cardiomyopathy | **31** | malignant tumor of pancreas |
| **11** | inflammatory bowel disease | **32** | down syndrome |
| **12** | preeclampsia | **33** | morbid obesity |
| **13** | crohn's disease | **34** | polycystic ovary syndrome |
| **14** | macular degeneration | **35** | epilepsy |
| **15** | osteoporosis | **36** | asthma, allergic |
| **16** | depression | **37** | allergic asthma |
| **17** | pancreatic cancer | **38** | eczema |
| **18** | psoriasis | **39** | acute myocardial infarction |
| **19** | arthropathy | **40** | mental retardation |
| **20** | asthma | **41** | atopic dermatitis |
| **21** | systemic lupus erythematosus (sle) | **42** | bacterial infection |

**Table S2. The DRD1s based on literature mining.**

| **Rank** | **DRD2s** | **Rank** | **DRD2s** |
| --- | --- | --- | --- |
| **1** | insulin resistance | **16** | acute myocardial infarction |
| **2** | obesity | **17** | macular degeneration |
| **3** | diabetic nephropathy | **18** | arthropathy |
| **4** | atherosclerosis | **19** | malignant tumor of pancreas |
| **5** | morbid obesity | **20** | hypoxia |
| **6** | senescence | **21** | hepatitis c infection |
| **7** | vitamin D | **22** | hepatitis c |
| **8** | depression | **23** | hepatocellular carcinoma |
| **9** | alzheimer's disease | **24** | carcinoma, hepatocellular |
| **10** | cardiomyopathy | **25** | breast cancer |
| **11** | polycystic ovary syndrome | **26** | anemia |
| **12** | osteoporosis | **27** | copd - chronic obstructive pulmonary disease |
| **13** | liver cirrhosis | **28** | rheumatoid arthritis |
| **14** | pancreatic cancer | **29** | colorectal cancer |
| **15** | nash | **30** | bacterial infection |

**Table S3. The parameter training results of different methods.**

| **DINet** | | | | |
| --- | --- | --- | --- | --- |
| **parameter** | **T1D(H_Dataset)** | **T2D(H_Dataset)** | **T1D(HMR_Dataset)** | **T2D(HMR_Dataset)** |
| 0.1 | 0.5362173 | 0.5302222 | 0.6118377 | 0.5577037 |
| 0.2 | 0.5365526 | 0.538963 | 0.6223452 | 0.5728889 |
| 0.3 | 0.5367762 | 0.5493333 | 0.6350324 | 0.574 |
| 0.4 | 0.5373351 | 0.5551111 | **0.6597362** | 0.5771852 |
| 0.5 | 0.5374469 | 0.5552593 | 0.6436396 | **0.5774074** |
| 0.6 | **0.5384529** | **0.5585185** | 0.605522 | 0.5753333 |
| 0.7 | 0.5384529 | 0.5573333 | 0.579924 | 0.5647407 |
| 0.8 | 0.5382294 | 0.5474074 | 0.5751733 | 0.5567407 |
| 0.9 | 0.5384529 | 0.5345185 | 0.5970266 | 0.5637778 |
| **DIoverlap_eDMN** | | | | |
| **expansion fold** | **T1D(H_Dataset)** | **T2D(H_Dataset)** | **T1D(HMR_Dataset)** | **T2D(HMR_Dataset)** |
| 1 | 0.6099933 | 0.5416296 | 0.6883523 | **0.6845185** |
| 2 | 0.6292198 | 0.6358519 | 0.7026604 | 0.6782222 |
| 3 | **0.6301140** | 0.6379259 | 0.7004248 | 0.6779259 |
| 4 | 0.6287726 | **0.6388148** | 0.6989716 | 0.6773333 |
| 5 | 0.6196065 | 0.6348148 | 0.6984127 | 0.6782222 |
| 6 | 0.6180416 | 0.6318519 | 0.6986363 | 0.6789630 |
| 7 | 0.6196065 | 0.6317037 | 0.6988598 | 0.6794074 |
| 8 | 0.6225129 | 0.6333333 | 0.7013190 | 0.6802963 |
| 9 | 0.6227364 | 0.6345185 | 0.7044489 | 0.6820741 |
| 10 | 0.6228482 | 0.6364444 | **0.7048960** | 0.6837037 |
| **DIconnectivity_eDMN** | | | | |
| **expansion fold** | **T1D(H_Dataset)** | **T2D(H_Dataset)** | **T1D(HMR_Dataset)** | **T2D(HMR_Dataset)** |
| 1 | 0.5960206 | 0.6325926 | 0.6959535 | 0.6989630 |
| 2 | 0.6128996 | 0.6469630 | 0.7028840 | 0.6997037 |
| 3 | 0.6186005 | **0.6520000** | **0.7097027** | 0.7094815 |
| 4 | **0.6212833** | 0.6469630 | 0.7050078 | **0.7131852** |
| 5 | 0.6136821 | 0.6413333 | 0.6993070 | 0.7077037 |
| 6 | 0.5974737 | 0.6334815 | 0.6885759 | 0.6961481 |
| 7 | 0.5682987 | 0.6280000 | 0.6787391 | 0.6878519 |
| 8 | 0.5427006 | 0.6185185 | 0.6644310 | 0.6754074 |
| 9 | 0.4755198 | 0.6118519 | 0.6516879 | 0.6641481 |
| 10 | 0.4804382 | 0.6050370 | 0.6396155 | 0.6543704 |

**Table S4. Top 20 eMNs (KF from large to small) of T1D-DRD1 connections under different SP thresholds.**

| **SP>0.1** | | | |
| --- | --- | --- | --- |
| **eMNs** | **KF** | **AF** | **SP** |
| GO:0060070_canonical Wnt signaling pathway | 41 | 237 | 0.172995781 |
| GO:0060828_regulation of canonical Wnt signaling pathway | 41 | 226 | 0.181415929 |
| GO:2000027_regulation of animal organ morphogenesis | 41 | 224 | 0.183035714 |
| GO:0000226_microtubule cytoskeleton organization | 40 | 244 | 0.163934426 |
| GO:0022604_regulation of cell morphogenesis | 40 | 240 | 0.166666667 |
| GO:0051090_regulation of DNA-binding transcription factor activity | 40 | 243 | 0.164609053 |
| GO:0050769_positive regulation of neurogenesis | 40 | 243 | 0.164609053 |
| GO:0051047_positive regulation of secretion | 40 | 243 | 0.164609053 |
| GO:0042391_regulation of membrane potential | 40 | 249 | 0.16064257 |
| GO:0002793_positive regulation of peptide secretion | 40 | 233 | 0.17167382 |
| GO:0016055_Wnt signaling pathway | 39 | 241 | 0.161825726 |
| GO:0198738_cell-cell signaling by wnt | 39 | 240 | 0.1625 |
| GO:0030111_regulation of Wnt signaling pathway | 39 | 226 | 0.172566372 |
| GO:0016050_vesicle organization | 39 | 224 | 0.174107143 |
| GO:0022412_cellular process involved in reproduction in multicellular organism | 39 | 229 | 0.170305677 |
| GO:0050804_modulation of chemical synaptic transmission | 39 | 240 | 0.1625 |
| GO:0051091_positive regulation of DNA-binding transcription factor activity | 39 | 235 | 0.165957447 |
| GO:0072001_renal system development | 39 | 243 | 0.160493827 |
| GO:0001822_kidney development | 39 | 234 | 0.166666667 |
| GO:0001655_urogenital system development | 39 | 222 | 0.175675676 |
| **SP>0.2** | | | |
| **eMNs** | **KF** | **AF** | **SP** |
| GO:0022613_ribonucleoprotein complex biogenesis | 34 | 167 | 0.203592814 |
| GO:0001501_skeletal system development | 34 | 170 | 0.2 |
| GO:0060485_mesenchyme development | 33 | 164 | 0.201219512 |
| GO:0072006_nephron development | 32 | 157 | 0.203821656 |
| GO:0030324_lung development | 30 | 148 | 0.202702703 |
| GO:0030323_respiratory tube development | 30 | 137 | 0.218978102 |
| GO:0030217_T cell differentiation | 30 | 150 | 0.2 |
| GO:0030278_regulation of ossification | 29 | 123 | 0.235772358 |
| GO:0060541_respiratory system development | 28 | 91 | 0.307692308 |
| GO:0072009_nephron epithelium development | 27 | 135 | 0.2 |
| GO:0060562_epithelial tube morphogenesis | 27 | 117 | 0.230769231 |
| GO:0055123_digestive system development | 27 | 133 | 0.203007519 |
| GO:0014706_striated muscle tissue development | 26 | 121 | 0.214876033 |
| GO:0001649_osteoblast differentiation | 26 | 98 | 0.265306122 |
| GO:0048638_regulation of developmental growth | 25 | 113 | 0.221238938 |
| GO:0010972_negative regulation of G2/M transition of mitotic cell cycle | 25 | 122 | 0.204918033 |
| GO:0050792_regulation of viral process | 25 | 113 | 0.221238938 |
| GO:0050678_regulation of epithelial cell proliferation | 25 | 115 | 0.217391304 |
| GO:0006397_mRNA processing | 24 | 97 | 0.24742268 |
| GO:1903311_regulation of mRNA metabolic process | 23 | 75 | 0.306666667 |
| **SP>0.3** | | | |
| **eMNs** | **KF** | **AF** | **SP** |
| GO:0060541_respiratory system development | 28 | 91 | 0.307692308 |
| GO:1903311_regulation of mRNA metabolic process | 23 | 75 | 0.306666667 |
| GO:1902105_regulation of leukocyte differentiation | 20 | 59 | 0.338983051 |
| GO:0052548_regulation of endopeptidase activity | 20 | 66 | 0.303030303 |
| GO:0007517_muscle organ development | 18 | 53 | 0.339622642 |
| GO:0071383_cellular response to steroid hormone stimulus | 18 | 47 | 0.382978723 |
| GO:0031100_animal organ regeneration | 18 | 56 | 0.321428571 |
| GO:0060537_muscle tissue development | 17 | 49 | 0.346938776 |
| GO:0009267_cellular response to starvation | 17 | 48 | 0.354166667 |
| GO:0003007_heart morphogenesis | 17 | 51 | 0.333333333 |
| GO:0021782_glial cell development | 16 | 52 | 0.307692308 |
| GO:0048545_response to steroid hormone | 16 | 53 | 0.301886792 |
| GO:0002521_leukocyte differentiation | 16 | 42 | 0.380952381 |
| GO:0071901_negative regulation of protein serine/threonine kinase activity | 16 | 42 | 0.380952381 |
| GO:0048771_tissue remodeling | 16 | 46 | 0.347826087 |
| GO:0042110_T cell activation | 16 | 52 | 0.307692308 |
| GO:0048732_gland development | 16 | 51 | 0.31372549 |
| GO:0043434_response to peptide hormone | 15 | 46 | 0.326086957 |
| GO:0051169_nuclear transport | 15 | 42 | 0.357142857 |
| GO:0036473_cell death in response to oxidative stress | 15 | 44 | 0.340909091 |
| **SP>0.4** | | | |
| **eMNs** | **KF** | **AF** | **SP** |
| GO:0062013_positive regulation of small molecule metabolic process | 15 | 35 | 0.428571429 |
| GO:1901653_cellular response to peptide | 14 | 30 | 0.466666667 |
| GO:0030218_erythrocyte differentiation | 14 | 35 | 0.4 |
| GO:0001659_temperature homeostasis | 14 | 32 | 0.4375 |
| GO:0032869_cellular response to insulin stimulus | 13 | 30 | 0.433333333 |
| GO:0043407_negative regulation of MAP kinase activity | 10 | 22 | 0.454545455 |
| GO:0032722_positive regulation of chemokine production | 10 | 24 | 0.416666667 |
| GO:0007173_epidermal growth factor receptor signaling pathway | 10 | 25 | 0.4 |
| GO:0019216_regulation of lipid metabolic process | 10 | 22 | 0.454545455 |
| GO:0008543_fibroblast growth factor receptor signaling pathway | 9 | 17 | 0.529411765 |
| GO:0030512_negative regulation of transforming growth factor beta receptor signaling pathway | 9 | 21 | 0.428571429 |
| GO:1901216_positive regulation of neuron death | 8 | 16 | 0.5 |
| GO:0010675_regulation of cellular carbohydrate metabolic process | 8 | 15 | 0.533333333 |
| GO:0046434_organophosphate catabolic process | 8 | 19 | 0.421052632 |
| GO:0051236_establishment of RNA localization | 7 | 17 | 0.411764706 |
| GO:0050772_positive regulation of axonogenesis | 7 | 14 | 0.5 |
| GO:0014003_oligodendrocyte development | 6 | 15 | 0.4 |
| GO:0050657_nucleic acid transport | 6 | 15 | 0.4 |
| GO:0048708_astrocyte differentiation | 6 | 11 | 0.545454545 |
| GO:0044364_disruption of cells of other organism | 6 | 11 | 0.545454545 |

From Table S4 we can see that when SP>0.2, the top eMNs have larger AFs; when SP>0.4, the top eMNs have smaller KFs, so we set the SP threshold to 0.3 to define specific eMNs.

**Table S5. Top 20 eMNs (KF from large to small) of T2D-DRD2 connections under different SP thresholds.**

| **SP>0.1** | | | |
| --- | --- | --- | --- |
| **eMNs** | **KF** | **AF** | **SP** |
| GO:0000226_microtubule cytoskeleton organization | 22 | 200 | 0.11 |
| GO:0051052_regulation of DNA metabolic process | 22 | 206 | 0.106796117 |
| GO:0016570_histone modification | 22 | 184 | 0.119565217 |
| GO:0198738_cell-cell signaling by wnt | 22 | 182 | 0.120879121 |
| GO:0016055_Wnt signaling pathway | 22 | 178 | 0.123595506 |
| GO:0090068_positive regulation of cell cycle process | 22 | 191 | 0.115183246 |
| GO:0048285_organelle fission | 22 | 209 | 0.105263158 |
| GO:0045787_positive regulation of cell cycle | 22 | 195 | 0.112820513 |
| GO:0045930_negative regulation of mitotic cell cycle | 22 | 187 | 0.117647059 |
| GO:0034660_ncRNA metabolic process | 21 | 176 | 0.119318182 |
| GO:0051260_protein homooligomerization | 21 | 165 | 0.127272727 |
| GO:0000082_G1/S transition of mitotic cell cycle | 21 | 180 | 0.116666667 |
| GO:0072331_signal transduction by p53 class mediator | 21 | 176 | 0.119318182 |
| GO:1901987_regulation of cell cycle phase transition | 21 | 196 | 0.107142857 |
| GO:0031396_regulation of protein ubiquitination | 21 | 167 | 0.125748503 |
| GO:1901990_regulation of mitotic cell cycle phase transition | 21 | 189 | 0.111111111 |
| GO:0016569_covalent chromatin modification | 20 | 144 | 0.138888889 |
| GO:0060070_canonical Wnt signaling pathway | 20 | 172 | 0.11627907 |
| GO:0044843_cell cycle G1/S phase transition | 20 | 166 | 0.120481928 |
| GO:0070646_protein modification by small protein removal | 20 | 187 | 0.106951872 |
| **SP>0.2** | | | |
| **eMNs** | **KF** | **AF** | **SP** |
| GO:0061138_morphogenesis of a branching epithelium | 14 | 67 | 0.208955224 |
| GO:0007626_locomotory behavior | 13 | 60 | 0.216666667 |
| GO:0001890_placenta development | 13 | 65 | 0.2 |
| GO:0007162_negative regulation of cell adhesion | 12 | 55 | 0.218181818 |
| GO:0001894_tissue homeostasis | 12 | 50 | 0.24 |
| GO:0060562_epithelial tube morphogenesis | 12 | 43 | 0.279069767 |
| GO:0034101_erythrocyte homeostasis | 12 | 55 | 0.218181818 |
| GO:0048469_cell maturation | 12 | 52 | 0.230769231 |
| GO:0009267_cellular response to starvation | 11 | 45 | 0.244444444 |
| GO:0007179_transforming growth factor beta receptor signaling pathway | 11 | 44 | 0.25 |
| GO:0042594_response to starvation | 11 | 43 | 0.255813953 |
| GO:0048762_mesenchymal cell differentiation | 11 | 42 | 0.261904762 |
| GO:0051100_negative regulation of binding | 11 | 53 | 0.20754717 |
| GO:0051047_positive regulation of secretion | 11 | 39 | 0.282051282 |
| GO:0030098_lymphocyte differentiation | 11 | 50 | 0.22 |
| GO:0001558_regulation of cell growth | 11 | 46 | 0.239130435 |
| GO:0006732_coenzyme metabolic process | 11 | 42 | 0.261904762 |
| GO:0032259_methylation | 10 | 41 | 0.243902439 |
| GO:0090287_regulation of cellular response to growth factor stimulus | 10 | 46 | 0.217391304 |
| GO:0019359_nicotinamide nucleotide biosynthetic process | 10 | 41 | 0.243902439 |
| **SP>0.3** | | | |
| **eMNs** | **KF** | **AF** | **SP** |
| GO:0071383_cellular response to steroid hormone stimulus | 10 | 32 | 0.3125 |
| GO:0006090_pyruvate metabolic process | 10 | 30 | 0.333333333 |
| GO:0090596_sensory organ morphogenesis | 9 | 30 | 0.3 |
| GO:0043401_steroid hormone mediated signaling pathway | 9 | 30 | 0.3 |
| GO:0016052_carbohydrate catabolic process | 9 | 29 | 0.310344828 |
| GO:0006397_mRNA processing | 9 | 24 | 0.375 |
| GO:0099173_postsynapse organization | 9 | 29 | 0.310344828 |
| GO:0001889_liver development | 9 | 25 | 0.36 |
| GO:0010770_positive regulation of cell morphogenesis involved in differentiation | 8 | 26 | 0.307692308 |
| GO:0048638_regulation of developmental growth | 8 | 23 | 0.347826087 |
| GO:1901292_nucleoside phosphate catabolic process | 8 | 26 | 0.307692308 |
| GO:0007584_response to nutrient | 8 | 26 | 0.307692308 |
| GO:0060541_respiratory system development | 7 | 18 | 0.388888889 |
| GO:0009755_hormone-mediated signaling pathway | 7 | 22 | 0.318181818 |
| GO:0046031_ADP metabolic process | 7 | 23 | 0.304347826 |
| GO:0090101_negative regulation of transmembrane receptor protein serine/threonine kinase signaling pathway | 6 | 17 | 0.352941176 |
| GO:0030518_intracellular steroid hormone receptor signaling pathway | 6 | 15 | 0.4 |
| GO:0048608_reproductive structure development | 6 | 16 | 0.375 |
| GO:0046394_carboxylic acid biosynthetic process | 6 | 20 | 0.3 |
| GO:0045639_positive regulation of myeloid cell differentiation | 6 | 18 | 0.333333333 |

From Table S5 we can see that when SP>0.1, the top eMNs have larger AFs (>100); when SP>0.3, the top eMNs have smaller KFs ($\leq10$), so we set the SP threshold to 0.2 to define specific eMNs.

**Table S6. The AUCs corresponding to different expanded gene numbers.**

| **the number of different expanded genes** | **the AUC of T1D** | **the AUC of T2D** |
| --- | --- | --- |
| 100 | 0.6008 | 0.4304 |
| 200 | 0.6808 | 0.6264 |
| 300 | 0.6792 | 0.6168 |
| 400 | 0.6792 | 0.6232 |
| 500 | 0.6816 | 0.6288 |
| 600 | 0.6816 | 0.6288 |
| 700 | 0.6816 | 0.6288 |
| 800 | 0.6816 | 0.6288 |
| 900 | 0.6816 | 0.6288 |
| 1000 | 0.6816 | 0.6288 |

We expanded (limited) the genes of diseases&vitamin D in steps of 100 (100, 200, …, 1000 ) to find the optimal expansion number. From Table S6 we can see that the optimal expansion number is 500.

**Table S7. The AUCs of different shortest path methods.**

| **shortest path method** | **the AUC of T1D** | **the AUC of T2D** |
| --- | --- | --- |
| $d_{s}(A,S)$ | 0.4700425 | 0.5333333 |
| $d_{c}(S,A)$ | 0.5342052 | 0.5441481 |
| $d_{c}(A,S)$ | 0.7023251 | 0.6885812 |

# Permutation training of MNs

1. For each diabetes-disease/vitamin D connection, we choose such eMNs: the numbers of diabetes and disease mapping genes in the corresponding MNs are both greater than 5.
2. The 255 eMN sets are permutated as follows：

Permutation 0: 1-255

Permutation 1: 2-255, 1

Permutation 2: 3-255, 1-2

Permutation 3: 4-255, 1-3

Permutation 4: 5-255, 1-4

Permutation 5: 6-255, 1-5.

1. Perform permutation training of eMN sets and choose the optimal result:

For DIoverlap_eDMN, Permutation 1 is the best (see Table S3);

For DIconnectivity _eDMN, Permutation 2 is the best (see Table S3).

# Random walk with restart

For a PPI network $G=(V,E)$, where $V$ represents the protein set and $E$ represents the interaction set. Let $A$ be the adjacency matrix of $G$, and $A(i,j)=1$ if protein $i$ interact with $j$, otherwise $A(i,j)=0$. Then$A$is normalized as follows

$A^{'}(i,j)=\frac{A(i,j)}{\sum_{k=1}^{n} A(k,j)}$, $n$ is the number of rows.

The random walker starts at a set of seed genes (e.g., the genes in MN, diabetes genes, disease genes), and finally the expanded gene set (e.g., the genes in eMN, expanded diabetes genes, expanded disease genes) can be obtained. The initial state $P_{0}$ is a column vector $P_{0}=[\psi_{1},\psi_{2}, ... , \psi_{n}]^{T}$, where $\psi_{i}$ is set to $\frac{1}{m}$ for the $m$ seed genes and 0 for other genes. It then randomly visit adjacent genes in every tick of time $(t\to t+1)$. The state probabilities$P_{t+1}$ at time $t+1$ is calculated as follows

$$P_{t+1}=(1-r)A^{'}P_{t}+rP_{0}$$

where $P_{t}$ is the probabilities at time $t$, $r$ is the restart probability (i.e., starting again from the seed genes). We set $r$ to be 0.7 as suggested by multiple previous studies [1]. This process is repeated until a steady-state is reached when the difference between $P_{t}$ and $P_{t+1}$ is smaller than 1e-6 as used by previous studies [2]. The steady-state probability of a gene represents how likely it will be visited from the seed genes and is used to rank the genes in $G$ (genes with higher steady probabilities will be ranked closer to the top).

[1] Shi H, et al. (2013). Walking the interactome to identify human miRNA-disease associations through the functional link between miRNA targets and disease genes. BMC systems biology. 7, 101. doi: 10.1186/1752-0509-7-101

[2] Hofree M, Shen JP, Carter H, Gross A, Ideker T. (2013). Network-based stratification of tumor mutations. Nature methods. 10, 1108–1115. doi: 10.1038/nmeth.2651
